# Validation of an elastic registration technique to estimate anatomical lung modification in Non-Small-Cell Lung Cancer Tomotherapy

**DOI:** 10.1186/1748-717X-6-31

**Published:** 2011-04-06

**Authors:** Elena Faggiano, Giovanni M Cattaneo, Cristina Ciavarro, Italo Dell'Oca, Diego Persano, Riccardo Calandrino, Giovanna Rizzo

**Affiliations:** 1 Istituto di Bioimmagini e Fisiologia Molecolare (IBFM), CNR, via Fratelli Cervi 93 Segrate (Milan), 20090, Italy; 2 Dept. of Biomedical Engineering, Politecnico di Milano, Milan, Italy; 3 Dept. of Medical Physics, Scientific Institute San Raffaele, Milan, Italy; 4 IRCCS Istituto Ortopedico Galeazzi, Milan, Italy; 5 Dept. of Radiotherapy, Scientific Institute San Raffaele, Milan, Italy; 6 Sciences Institute, National University of General Sarmiento, Buenos Aires, Argentina; 7 Dept. of Nuclear Medicine, Scientific Institute San Raffaele, Milan, Italy

## Abstract

**Background:**

The study of lung parenchyma anatomical modification is useful to estimate dose discrepancies during the radiation treatment of Non-Small-Cell Lung Cancer (NSCLC) patients. We propose and validate a method, based on free-form deformation and mutual information, to elastically register planning kVCT with daily MVCT images, to estimate lung parenchyma modification during Tomotherapy.

**Methods:**

We analyzed 15 registrations between the planning kVCT and 3 MVCT images for each of the 5 NSCLC patients. Image registration accuracy was evaluated by visual inspection and, quantitatively, by Correlation Coefficients (CC) and Target Registration Errors (TRE). Finally, a lung volume correspondence analysis was performed to specifically evaluate registration accuracy in lungs.

**Results:**

Results showed that elastic registration was always satisfactory, both qualitatively and quantitatively: TRE after elastic registration (average value of 3.6 mm) remained comparable and often smaller than voxel resolution. Lung volume variations were well estimated by elastic registration (average volume and centroid errors of 1.78% and 0.87 mm, respectively).

**Conclusions:**

Our results demonstrate that this method is able to estimate lung deformations in thorax MVCT, with an accuracy within 3.6 mm comparable or smaller than the voxel dimension of the kVCT and MVCT images. It could be used to estimate lung parenchyma dose variations in thoracic Tomotherapy.

## Background

Helical Tomotherapy (HT) is an approach that combines Intensity-Modulated Radiation Therapy delivery with built-in image guidance using megavoltage CT scans (MVCT) [1]. The technique uses a binary multileaf collimator able to create very sharp dose distributions around the target volumes.

In HT, daily MVCT scans of the patient in the treatment position are available with acquisition geometry identical to treatment delivery geometry. In clinics, MVCT images are primarily used for patient setup verification [2]. For this purpose, the MVCT images are rigidly registered with the kVCT image and the patient is then automatically repositioned for treatment delivery according to rigid registration parameters. However, during radiation treatment, patients may undergo significant anatomical changes. In the case of Non-Small-Cell Lung Cancer (NSCLC), lung parenchyma can significantly modify its volume and shape [3]. As a direct consequence, in lungs, dose discrepancies can occur between the planned cumulative dose distribution and the actual cumulative dose [4]. This is a major point in lung cancer as lung parenchyma is one of the most radiosensitive healthy tissues in the thorax and the cumulative dose represents the correct value to be used in relating dosimetric indices with treatment outcome [5].

To analyze and study the anatomical changes of lung parenchyma due to radiation therapy, and to calculate the corresponding accumulated dose, rigid registration methods are not sufficient; the introduction of deformable registration methods has therefore been fundamental [6]. In the field of HT, very few studies on deformable registration methods between kVCT and MVCT have been addressed. Deformation was first introduced in 2006 by Lu and coworkers, and applied to various anatomical districts (head-neck, chest, lower abdomen) [7]. The Lu's method was based on a free deformation model in which every voxel was free to move. The sum of square difference was used as similarity measure to match the images and the smoothness of the deformation as a constraint. The problem was then represented as a set of nonlinear elliptic partial differential equations through calculus of variations and solved with a Gauss-Siedel finite difference scheme in a multi-resolution framework. The same registration approach was also applied later in head-and-neck cancer patients treated with HT [8,9]. Recently, a different registration procedure consisting in a multiple preprocessing step and a two-step optical flow deformable registration method was proposed to register abdominal MVCT and kVCT images [10].

Concerning the thoracic district, deformable image registration is widely used typically in 4D-CT and respiratory-correlated CT protocols [11,12], to correctly model respiratory motion. However, it is recognized that most facilities currently do not have access to methods that explicitly account for respiratory motion, and that respiratory management methods are not required for all patients irradiated for thoracic tumors [13]. Nowadays, all vendors provide basic equipment for image guided radiotherapy using on-board volumetric X-ray imaging with continuous radiography and (slow) gantry rotation for back-projection reconstruction, but only major research groups have implemented daily respiratory correlated 4D-CT [14,15]. In free-breathing clinical protocols only Guckenberger *et al. *[16] have studied the performance of their proposed surface-based deformable image registration method to register kVCT to kVCT images. Authors outlined the importance of registration between images taken during the course of radiotherapy treatment; in fact, in this case, registration is significantly more diffcult than in respiratory correlated images, because of drastic anatomical changes due to tumor regression, weight loss of patients and variations of pleural effusion and atelectasis [16]. In this context, studying the application of deformable registration for kVCT and MVCT images acquired during free-breathing in clinical HT protocols remains of major interest. However, to the best of our knowledge, only Lu's first study [7] approached the registration of thoracic free-breathing kVCT and MVCT images; they analysed the efficacy of their method on two lung-cancer patients evaluating the registration results in terms of qualitative analysis and correlation coefficient comparisons between the rigid and the elastic approach.

The aim of this work is to propose and validate a different technique for the elastic registration of kVCT and MVCT thoracic images acquired during free-breathing in clinical HT protocols. The proposed method consists in a rigid body deformation combined with a cubic B-spline deformation model in which only a regular grid of control points is free to move [17]. The mutual information is used as similarity criterion to match the images [18], making the method capable of working with multi-modal images. A four steps multi-resolution strategy is used to solve the registration problem with a limited-memory quasi-Newton algorithm as optimizer.

This approach, originally proposed for positron emission tomography and CT registration by Mattes *et al. *[18], was extensively used for medical image registration [17,19]; however it has never been studied in HT thoracic application before. Here, we adapted the method to the specific thoracic HT application and evaluated its accuracy to NSCLC patients, to investigate whether the technique is adequate to detect lung deformations during and following radiotherapy.

## Methods

### Patient dataset

The study included 5 patients treated for locally advanced NSCLC, stage III A - III B on an HT unit (HiArt2 Tomotherapy, Madison, Wisconsin). Patients were treated with radiation therapy alone, due to medical status, with radical intent. The chosen patient population presented large heterogeneity with respect to the effects induced by HT: mediastinum shift due to tumor regression, increased pleural effusion and atelectasis, weight loss. The treatment schedule was 2.5 Gy for 25 days of treatment (1 fraction/day, 5 fractions/week), for a total dose of 62.5 Gy. The protocol was approved by the Local Ethics Committee. Written, informed consent to treatment was obtained from all patients.

Registration between the planning kVCT and 3 daily MVCT images of each patient was analyzed for a total of 15 studies; we considered one MVCT scan at the beginning of treatment, one in the middle and one at the end, in order to account for different stages of anatomical deformation induced by HT treatment. The kVCT images of all patients were acquired with an MDCT scanner (LightSpeed, GE Medical System, Milwaukee, USA). The number of slices in these images ranged from 84 to 99, and each slice was 512 × 512 pixels with voxel size equal to 0.976 × 0.976 × 3.27 mm^3^. For patients treated with dose per fraction lower than 5 Gy, or in the presence of limited tumor movement, our standard imaging protocol included a free-breathing helical CT covering all the thorax. These images were used to calculate the radiotherapy plan, dose distribution and dose volume histogram both for target volume and Organs at Risk.

The daily MVCT images of all patients were acquired using the on-board HiArt2 CT scanner of the HT unit. MVCT images were acquired prior to each treatment fraction and were clinically used for patient repositioning. Each slice was 512 × 512 pixels with variable voxel size from 0.754 × 0.754 × 4 mm^3 ^to 0.754 × 0.754 × 6 mm^3^. MVCT delivers higher dosages to the patient with lower image quality than diagnostic kVCT. The typical patient MVCT imaging dose was in the range 1.0-2.0 cGy [20]. MVCT images were relatively smaller and were included in the reference kVCT image space, as MVCT acquisition was performed paying attention to patient irradiation sparing. The number of slices was different for each patient on different days, ranging from 21 to 39 for a voxel axial dimension of 4 mm, and from 8 to 18 for a voxel axial dimension of 6 mm. The MVCT imaging system acquires scans under free-breathing conditions with a slow spiral (10 s/gantry rotation); on average, multiple respiration phases are recorded per slice.

### Image registration

A requirement of image registration is that the same physical volume extent is imaged in the two studies to be registered. As kVCT and MVCT presented differences in image extent, we introduced a pre-processing step to deal with them. In details image pre-processing included the following steps: (1) the treatment couch was manually deleted from kVCT and MVCT images, (2) voxels not belonging to the patient body were deleted from both kVCT and MVCT to exclude most of the voxels, which do not contain useful information for the registration process, (3) kVCT slices were also cropped along the axial direction to match MVCT slices, in order to avoid a great number of spatial samples falling out of the MVCT domain. If the acquired MVCT had a different field of view, we made different reductions for each MVCT. After these pre-processing steps, kVCT and MVCT datasets imaged the same anatomical volumes.

Registration was applied between MVCT images at each stage and the kVCT images chosen as reference. The spatial transformation was modeled as a sum of a global rigid transformation to correct the global misalignment and a local elastic deformation. Both transformations were estimated using the similarity measure of mutual information (MI) in the form proposed by Mattes *et al. *[18] as the minimization criterion. We implemented our code within the Insight Segmentation and Registration Toolkit (ITK) [21], because of its efficiency and user-friendliness.

Rigid transformation was found using a three-level multi-resolution strategy creating an image pyramid with the suggested ITK schedules as down-sampling parameters [21]. Moreover, the optimizer convergence tolerance (step length) was changed during iterations (step length set equal to 10^2^, 5·10^3^, 2.5·10^3 ^for the first, second and third level).

Elastic deformation was modeled using free-form deformations based on cubic B-splines [17] defined on a regular grid of control points. In order to avoid local minima and to decrease computation time we adopted a multi-resolution strategy of 4 iterative steps for both the deformation grid and the images with a multi-resolution parameter settings listed in Table 1. Concerning multi-resolution of the grid, the 4 steps were characterized by a progressively increased number of control points. The grid resolution was chosen to tailor the registration method on the specific thoracic application. Specifically, in the first step a grid resolution of about 96 *mm *was set, while the last step used a resolution of 30 *mm *in each direction [22]. Concerning the multi-resolution of the images, a Gaussian blurring was applied with a kernel that narrowed as multi-resolution proceeded [18]. The Gaussian blurring in the axial direction was modified to take into account different MVCT axial dimensions (see Table 1 for the Gaussian rule). Moreover, we increased the percentage of voxels used to estimate the mutual information as multi-resolution proceeded. As regards the adopted optimization algorithms, L-BFGS-B optimizer was used [21] varying the tolerance of the termination criterion as suggested in [18]. A typical registration takes approximately 30 min on a 2.26 GHz Intel(R) Xeon(R) processor, with 6 GB RAM.

**Table 1:** Parameter settings for the elastic method.

parameter		*L*_1_	*L*_2_	*L*_3_	*L*_4_
Gaussian kernel (pixels)	If *z *dimension ≥ (*x *dimension)	16/16/16	8/8/8	2/2/2	0/0/0
	If *z *dimension < (*x *dimension)	16/16/8	8/8/4	2/2/2	0/0/0
percentage voxels used		0.8	3.4	9.3	19.7
L-BFGS-B tolerance		10^-5^	10^-6^	10^-7^	10^-8^

### Assessment of the registration accuracy

The accuracy of the registration technique was first evaluated qualitatively. Two authors (G.M.C. and I. D.), both radiotherapy image experts, evaluated image-matching accuracy in each patient and each pair of kVCT to MVCT registrations by visual inspection. Quantitative assessment of accuracy was performed in terms of correlation coefficient (CC) and Target Registration Error (TRE) estimated by anatomical landmarks. CC can be used as a global index of the registration performance [23], while TRE gives a global measure of registration accuracy [24]. Finally, to specifically evaluate registration accuracy in lungs, we performed a lung volume correspondence analysis.

#### Correlation coefficient

Correlation coefficient (CC) is defined as:(1)

where *x_i _*is the intensity of the *i *- *th *voxel in the fixed image and *y_i _*is the intensity of the corresponding voxel in the registered image;  and  are the mean intensity of the fixed and the registered image, respectively. If there is a linear correlation between the two image intensity values, the absolute value of CC is equal to 1. CC coefficient was widely used to validate deformable registration algorithms and could be considered a standard index in accuracy evaluation of registration methods, when dealing with similar image modalities [7,25].

We determined CC in the overlap of both the two images excluding a border of 30 × 30 voxels in x,y directions and the first and last 2 planes in z direction. This was done to remove areas interpolated from the external of the image volume, thus containing not reliable information.

#### Target Registration Error

On kVCT and MVCT images the two experts identified corresponding anatomical landmarks by mutual consensus. Several markers were detected in specific areas: rib, breast-bone, carina, bronchial bifurcation, nipple, vertebral body, aortic arch and lung apex. Other markers were patient-specific (calcifications or easily recognizable anatomical details). Only a subset of the detected markers was visible for each MVCT (ranging from 2 to 6), because of the low contrast and axial dimension of MVCT images. Only the visible MVCT markers were considered in the TRE analysis. The landmark positions (*x_i_*, *y_i_*, *z_i_*) identified on kVCT images were moved according to the spatial transformation found by the rigid and elastic registration algorithms in order to obtain their transformed positions ()relative to the MVCT spatial reference system:(2)

Registration accuracy was defined, in terms of TRE, by the residual misalignment between () and the landmark positions directly detected by the experts () on MVCT images:(3)

#### Lung volume correspondence analysis

For lung volume correspondence analysis, corresponding lung surfaces had to be estimated from kVCT and rigidly and elastically registered MVCT images. To do this, a region growing algorithm, implemented in a commercial software package (Analyze 4.0, Biomedical Imaging Resource, Mayo Clinic, Rochester, MN) was applied slice-by-slice for contour identification to both the kVCT and the MVCT images. The region growing algorithm required a lower and upper intensity thresholds: we set these values at -1000 HU (Hounsfield unit) and -500 HU, respectively, for both kVCT and MVCT images and for each patient [26]. This procedure corresponds to the standard procedure adopted in our institute, and allows proper extraction of lung contours as verified by human observers. For each slice, a binary image representing the lung structure was created by setting the voxels inside the identified contours to 1 and the voxels outside to 0. The lung volume was then created by piling up the binary slices. After volumes were constructed, the volume error, the centroid error and a matching similarity index were used to compare how well the two corresponding lung volumes matched each other after registration. The volume error (*V_E_*) was calculated by comparing the volume in mm^3 ^of the kVCT left/right lung (*V*^CT^) with the rigidly and elastically registered MVCT volumes () [27]:(4)

The centroid error (*C*_E_) was calculated by comparing the centroids of the same volumes (*C*^CT ^and ):(5)

The Jaccard index (JAC) was used as the matching similarity index [28]. JAC indicates the overlapping ratio between the kVCT volume set *R*^CT ^and the registered volume set :(6)

If the two volume sets are identical, JAC is equal to one; if they have no common region, JAC is equal to zero. The described measures were always used to compare the lung sub-region that was imaged in both KVCT and MVCT: in general this region did not cover the entire lung volume because, as mentioned previously, the MVCT was acquired by covering the smallest possible lung region for patient irradiation sparing.

Statistical significance of the differences between rigid and elastic indices was assessed using Wilcoxon signed rank test as implemented in MATLAB 64bit (R2009b, The MathWorks, Natick, MA).

## Results

All patients presented anatomical changes during the course of therapy as shown by TRE values and lung volume correspondence indices calculated after the sole rigid registration (Table 2, 3 and 4). For example large TREs were found in patient 4, who presented a significant weight loss and in patient 1 because of mediastinum shift (Table 2). Furthermore patient 1 presented a mediastinum shift during the course of therapy and an increasing atelectasis of the left lung with a consequent decrease of the left lung volume and a small increase of the right lung volume (see Table 3 and 4). The increase in left and right volumes in patient 3 and 4 was due to the resolved large pleural effusion. Patient 3, 4 and 5 presented major changes in lung anatomy, due to tumor regression.

**Table 2:** Target Registration Error.

		kVCT/1st MVCT			kVCT/2nd MVCT			kVCT/3rd MVCT			mean values	
		#	TRE(mm)		#	TRE(mm)		#	TRE(mm)		TRE(mm)	
patient	registration	mrks	mean ± SD	max	mrks	mean ± SD	max	mrks	mean ± SD	max	mean ± SD	max
1.	rigid	4	5.16 ± 1.16	6.56	4	9.17 ± 5.46	16.72	4	6.43 ± 2.65	10.24	6.92 ± 2.05	16.72
	elastic		2.42 ± 0.73	3.35		4.18 ± 2.17	7.28		4.11 ± 2.17	7.20	3.57 ± 1.00	7.28
2.	rigid	6	3.2 ± 1.60	5.26	6	4.2 ± 2.19	6.42	4	4.4 ± 4.13	10.39	3.93 ± 0.64	10.39
	elastic		3.46 ± 0.92	4.67		3.02 ± 1.26	4.96		4.29 ± 3.58	9.63	3.59 ± 0.64	9.63
3.	rigid	5	6.41 ± 2.68	10.17	4	2.93 ± 0.90	4.27	4	4.44 ± 1.66	6.81	4.59 ± 1.75	10.17
	elastic		5.51 ± 2.44	8.51		2.81 ± 1.06	4.09		4.2 ± 1.45	6.15	4.17 ± 1.35	8.51
4.	rigid	5	5.39 ± 2.51	8.33	5	7.95 ± 2.62	11.13	4	9.86 ± 5.34	14.46	7.73 ± 2.24	14.46
	elastic		3.82 ± 2.89	8.76		4.83 ± 3.73	11.22		5.1 ± 2.87	8.35	4.58 ± 0.67	11.22
5.	rigid	5	3.08 ± 1.13	4.57	4	2.87 ± 1.38	3.83	2	2.99 ± 1.64	4.15	2.98 ± 0.11	4.57
	elastic		3.16 ± 0.94	4.55		2.14 ± 1.00	3.63		2.52 ± 2.13	4.02	2.61 ± 0.52	4.55

Number of markers (# mrks), average and maximum TRE values are shown.

**Table 3:** Volume error (*V*_E_), centroid error (*C*_E_) and JAC index for right lung.

		kVCT/1st MVCT			kVCT/2nd MVCT			kVCT/3rd MVCT		
patient	registration	*V*_E _%	*C*_E_	JAC	*V*_E _%	*C*_E_	JAC	*V*_E _%	*C*_E_	JAC
1.	rigid	-8.07	3.10	0.89	11.30	3.98	0.84	-8.54	4.73	0.87
	elastic	-0.69	0.77	0.95	0.62	1.22	0.95	-0.19	0.53	0.95
2.	rigid	4.44	1.43	0.90	10.12	2.71	0.87	-2.17	2.82	0.71
	elastic	2.03	0.53	0.93	4.78	0.78	0.93	1.61	0.32	0.93
3.	rigid	3.33	2.89	0.88	3.73	1.77	0.88	-3.73	1.77	0.91
	elastic	2.09	0.28	0.95	1.59	0.33	0.95	-0.41	0.39	0.96
4.	rigid	-0.55	0.95	0.90	-1.44	1.30	0.89	-11.14	1.56	0.82
	elastic	0.48	0.18	0.93	-1.15	0.28	0.94	-3.11	0.88	0.93
5.	rigid	7.64	3.01	0.89	0.90	2.68	0.91	-16.16	6.12	0.80
	elastic	3.45	0.73	0.94	-0.72	0.50	0.96	-5.23	5.46	0.87

**Table 4:** Volume error (*V*_E_), centroid error (*C*_E_) and JAC index for left lung.

		kVCT/1st MVCT			kVCT/2nd MVCT			kVCT/3rd MVCT		
patient	registration	*V*_E _%	*C*_E_	JAC	*V*_E _%	*C*_E_	JAC	*V*_E _%	*C*_E_	JAC
1.	rigid	24.85	7.35	0.72	-17.87	9.76	0.76	45.14	7.86	0.54
	elastic	1.97	0.40	0.95	-0.81	0.14	0.96	3.10	0.84	0.92
2.	rigid	-5.14	1.22	0.90	-5.55	2.05	0.89	2.64	0.93	0.89
	elastic	0.25	0.39	0.94	1.49	0.26	0.95	1.11	0.76	0.92
3.	rigid	-1.97	2.15	0.80	-4.53	2.58	0.87	-17.38	5.15	0.81
	elastic	1.99	1.94	0.89	0.01	2.11	0.90	-6.82	3.23	0.88
4.	rigid	-0.80	2.00	0.91	-5.42	2.14	0.90	-10.69	4.06	0.84
	elastic	0.80	0.06	0.95	-0.59	0.37	0.96	-2.08	0.56	0.95
5.	rigid	2.35	0.89	0.92	4.60	0.96	0.93	4.26	0.99	0.90
	elastic	2.23	0.16	0.95	0.49	0.09	0.96	2.05	1.52	0.94

**Figure 1:**
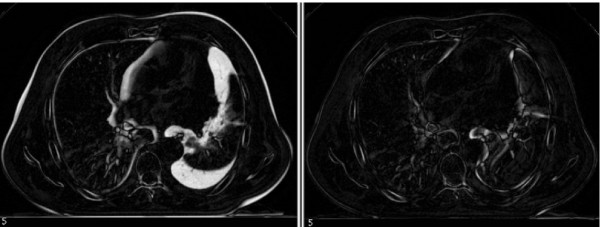
**Image difference between kVCT and MVCT phase 3 in patient 4**. Left: rigid registration, right: elastic registration. In this image pixel intensity is proportionally related to the degree of mismatching between images (black values matching, white values mismatching). White areas indicating mismatching between kVCT and registered MVCT image due to large pleural effusion in patient 4, were recovered by the elastic registration.

The radiotherapy experts judged image elastic registration adequate in all cases to correctly follow anatomical variations between kVCT and MVCT, and among different MVCT acquisitions. The good performance of elastic deformation can also be appreciated in Figure 1, which shows the difference-images obtained using kVCT and MVCT after rigid and elastic registration in a patient with large pleural effusion: elastic registration could take into account the pleural effusion and allowed good superposition of all areas still mismatched after rigid registration. Similar results were obtained for each patient in each kVCT/MVCT registration.

Table 5 summarizes registration results in terms of CC before and after elastic registration: for all patients CC values significantly increased (*p *= 10^-5^, Wilcoxon signed rank test) after elastic registration and were between 0.97 - 0.99, thus proving good recover of deformed structures.

**Table 5:** Correlation coefficients (**CC**) after rigid and elastic registration.

	patient and session															mean ± SD
	**1/1**	**1/2**	**1/3**	**2/1**	**2/2**	**2/3**	**3/1**	**3/2**	**3/3**	**4/1**	**4/2**	**4/3**	**5/1**	**5/2**	**5/3**	
rigid	0.92	0.91	0.88	0.97	0.97	0.96	0.90	0.96	0.90	0.92	0.92	0.86	0.95	0.96	0.94	0.93 ± 0.03
elastic	0.98	0.98	0.98	0.99	0.99	0.98	0.98	0.98	0.97	0.98	0.97	0.97	0.98	0.98	0.97	0.98 ± 0.01

Quantitative values of image registration accuracy in terms of TRE are shown in Table 2. Elastic registration performed well in the majority of cases, leading to a significant average and maximum TRE reduction (*p *= 0.0015 and *p *= 10^-4 ^respectively, Wilcoxon signed rank test), especially when large average TRE was present before elastic registration. It should be noted that, after elastic registration, average TRE remained comparable to, or often smaller than, voxel resolution.

**Figure 2:**
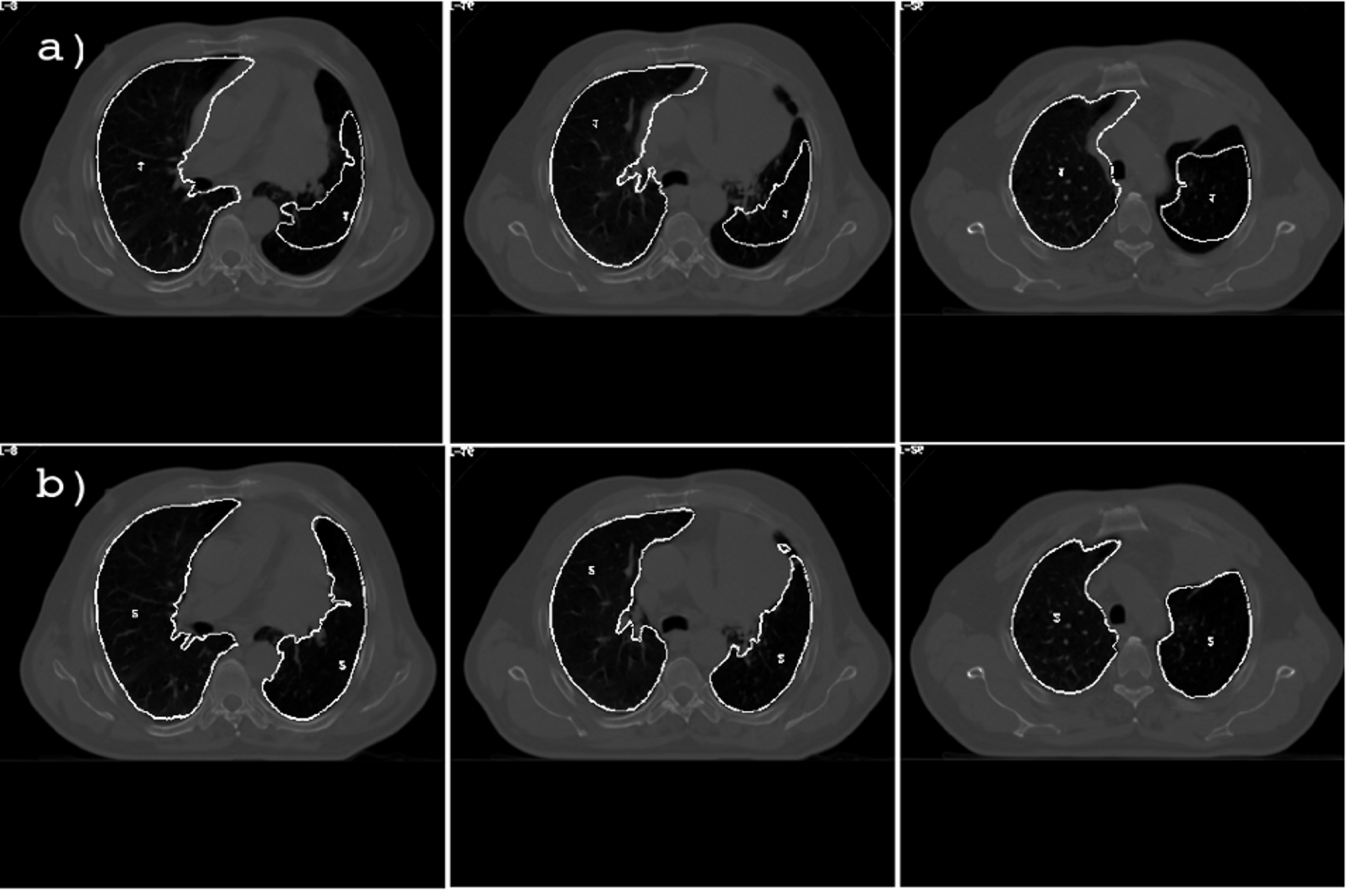
**Comparison of lung contours correspondence**. kVCT slices for patient 1 with superimposed lung contours extracted from the MVCT after a sole rigid realignment (a) and the elastic method (b). Elastic lung contours well delineate the lung volume on kVCT image while realigned MVCT contours are very different because of mediastinum shift and atelectasis.

Regarding the lung volume correspondence analysis, Figure 2 shows, for a qualitative evaluation, three kVCT slices of patient 1 with superimposed contours delineated on the MVCT obtained after sole rigid realignment and after the application of the elastic algorithm. This patient experienced a large mediastinum shift accompanied with large atelectasis. These major anatomical modifications were clearly recovered by elastic registration: elastic contours are well superimposed onto the kVCT lungs, while before registration MVCT lung was substantially different from kVCT lung. A qualitative goodness of lung superimposition obtained after elastic registration occurred in all cases.

**Figure 3:**
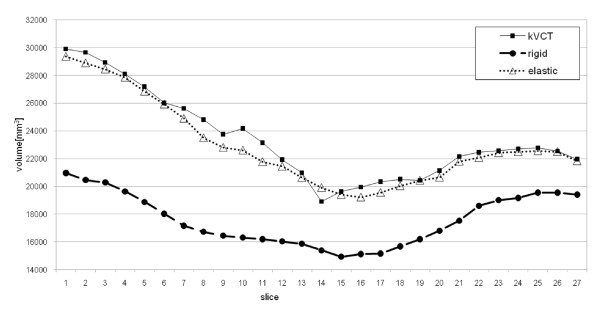
**Slice-by-slice volume comparison in left lung (patient 1)**. kVCT lung volume (full square), rigidly realigned MVCT lung volume (full circle) and elastically registered MVCT lung volume(triangle). The rigid volume trend demonstrated volume reduction in MVCT due to large left lung atlectasis increase and mediastinum shift in the direction of left lung. Elastic volume trend well fitted the kVCT trend demonstrating good recovering of deformation.

Comparison between a lung volume extracted from kVCT images, the corresponding rigidly realigned and elastically registered MVCT images is graphically presented in Figure 3 (again patient 1). This is an easy and effective visualization to appreciate the performance of the registration method in presence of large left lung atelectasis and mediastinum shift in the direction of the left lung: while the rigidly realigned MVCT lung presented a lung volume systematically smaller than the initial kVCT lung volume, after elastic registration volume values were similar to the kVCT ones. Quantitatively, in this case, kVCT lung volume was 632.39 cm^3 ^and MVCT volume was 475.22 cm^3^, with a volume difference of 157.17 cm^3^; elastic registration recovered the lung volume well (elastic MVCT volume was 619.95 cm^3 ^with a residual volume error of 12.44 cm^3^).

Table 3 and Table 4 show the results of the quantitative analysis of lung volumes, in terms of *V*_E_, *C*_E _and JAC for right and left lung respectively. *V*_E _in absolute value was between 0.19% - 5.23% for right lung and between 0.01% - 6.82% for left lung, while, before elastic registration, a significant higher error was present (between 0.55% - 16.16% for right lung and between 0.8% - 45.14% for left lung, *p *= 10^-5 ^and *p *= 10^-4 ^right and left lung respectively, Wilcoxon signed rank test). Considering both lungs and the average value over the three MVCT sessions, an average volume error of 1.78% was found after elastic registration starting from an average error of 8.22%. *C*_E _was between 0.18 - 5.46 mm for right lung and between 0.06 - 3.23 mm for left lung; considering both lungs and the average value over the three MVCT sessions, an average error of 0.87 mm was found. Also in these cases, elastic registration significantly recovered (*p *= 10^-5 ^and *p *= 10^-4 ^right and left lung respectively, Wilcoxon signed rank test) volume discrepancies induced by HT as estimated by rigid realignment (between 0.95 - 6.12 mm for right lung and between 0.89 - 9.76 mm for left lung with an average error over the three MVCT sessions of 3.03 mm). After elastic registration JAC demonstrated a good matching in lung structure with high values (between 0.87 - 0.96 for right lung and between 0.88 - 0.96 for left lung) significantly increased with respect to JACs obtained with rigid registration (between 0.71 - 0.91 in right lung and between 0.54 - 0.93 in left lung, *p *= 10^-5 ^and *p *= 10^-5 ^right and left lung respectively, Wilcoxon signed rank test). In summary, in all cases lungs changed during the course of treatment and the performed elastic registration well estimated these changes with small residual errors.

## Discussion

In this study we evaluated an elastic registration method based on B-spline free-form deformation and mutual information metric for the registration of thoracic free-breathing MVCT to kVCT images of NSCLC patients. This approach, already very popular in the field of image registration, has never been studied in this specific context. The accuracy of the method was systematically evaluated by means of CC, TRE and lung volume correspondence analysis.

Our results show that all five patients in the study underwent significant anatomical changes during the course of therapy. Weigh loss, pleural effusion, atelectasis and free-breathing acquisition involved numerous differences in MVCT images with respect to kVCT planning images and high TREs, volume errors and centroid errors before elastic registration are a measures of these differences. Elastic registration was able to significantly reduce these sources of errors. In particular, CC values were always found to be high and registration accuracy was good, with small TRE values demonstrating a registration accuracy comparable to voxel resolution. Moreover lung volume variations were well detected by the elastic algorithm with a residual volume error ranging from 0.01% to 6.82%. The goodness of the elastic approach was also confirmed in terms of residual centroid shift (almost always smaller that 0.9 mm) and JAC index, which was always high with values higher than 0.87.

In the recent literature there has been considerable examination of HT methodology applied to lung cancer [29-31] and a very often raised point is the importance of the good anatomical correspondence of tissues in the spatial reference systems defined during the radiotherapy planning and each HT irradiation session. In fact, this is important for the control of dose delivery to minimize side effects, also having prospectively in mind adaptive HT protocols [26,32]. In this context, the study of lung deformation is very important because the actual accumulated dose in lung parenchyma, which can be correctly calculated only when based on the accurate knowledge of the spatial position covered by the lungs, is an important index used to decide when and how the radiotherapy plan should be modified [4].

A thoroughly investigated aspect concerns lung registration in 4D protocols using respiratory gating acquisition approaches [11,12,22]. However, clinical HT instruments are still not equipped for gating, and irradiation is usually carried out using standard free-breathing respiration protocols [13].

Notwithstanding this evidence, as far as we know, this work presents the first systematic evaluation of the registration accuracy of an elastic method to register free-breathing kVCT and MVCT images in the lung district in HT clinical protocols. Before this, only Lu *et al. *[7] proposed a deformable registration approach in HT free-breathing lung clinical protocols that used an intensity-based method adopting the sum of square distance as the similarity measure. In that pioneering paper, the registration was performed only in two patients with lung cancer and was evaluated only using correlation coefficient comparisons between rigid and elastic approach. In our work the accuracy evaluation was thoroughly analyzed on 15 kVCT to MVCT registration studies, relative to 5 patients who presented large variety with respect to anatomical modifications due to HT. We evaluated the method not only using CC and TRE to assess the global performance of elastic approach, but also introducing a lung correspondence analysis to study registration performance in lung. Very recently Guckenberger *et al. *[16] also studied elastic registration in free-breathing lung clinical protocols using a surface-based deformable registration method to perform kVCT to kVCT registration. Comparing our study with their work, our results were similar or better than their results in terms of both CC and TRE with the additional advantage of using an intensity based method, which doesn't require surface segmentation as in surface-based registration.

In summary, our results showed that the proposed elastic registration method is accurate for kVCT-MVCT lung registration in free-breathing HT protocols. Although the performance of our method was thoroughly evaluated in a set of 15 registrations of 5 patients representing a variety of conditions, a confirmation in a larger number of cases could further reinforce our results. The good performance of our method suggests that it could be used effectively for the analysis of lung deformations in the context of HT NSCLC protocols and, prospectively, to obtain an accurate estimation of cumulative dose distribution in lungs [26]. In NSCLC radiotherapy, patients may undergo clinically significant symptomatic radiation pneumonitis in approximately 5 - 50% of cases. The rate and severity of radiation-induced sequelae are related to dosimetric indices derived from the lung dose-volume histogram [33]. For instance, the percentage of lung parenchyma receiving more than 20 Gy is associated with a radiation pneumonitis risk, which is low or unacceptable if the percentage is < 20% or > 35%, respectively. Due to the changes in normal tissue anatomy during treatment, the plans defined on the basis of pre-HT imaging may not accurately reflect the degree of normal lung exposure. Thus, the possibility of calculating accumulated dose-volume distributions corrected for lung anatomical modifications in HT treatment of NSCLC can lead to two important benefits in lung RT: (1) changes in normal tissue functionality can be related to the true accumulated dose with important improvements in the comprehension of radiation effect mechanisms in normal tissue [5] (2) it can open the basis for an adaptive approach: if the dosimetric parameter surrogate of lung side effects is approaching a "not acceptable" value, the RT plan can be re-evaluated. In the perspective of adaptive radiotherapy, the evaluation of our registration method, here focused on lung parenchyma, should be also extended to the tumor volume, in order to thoroughly assessed registration accuracy. In the case of locally advanced NSCLC, the tumor delineation on MVCT scans presents some difficulties; therefore the validation of this method in following changes in tumor size/location during Tomotherapy is currently underway at our institution and will be described in further works.

## Conclusion

In this work, we proposed and validated a method based on free-form deformation and mutual information to perform elastic registration for treatment planning kVCT images and daily MVCT images in NSCLC patients using free-breathing acquisition protocols. The systematic evaluation of registration accuracy to detect lung anatomical variations suggests the applicability of this registration method as an accurate tool to estimate lung parenchyma dose variations in thoracic Tomotherapy.

## Competing interests

The authors declare that they have no competing interests.

## Authors' contributions

All authors read and approved the final manuscript.

EF implemented elastic registration and analyzed data, contributed to draft and revised the manuscript. GMC designed the patient study and participated in the revision of the manuscript. CC implemented rigid registration and participated in the data analysis. IDO participated in design of the patient study and in data analysis. DP contributed in elastic registration setup. RC participated in the data analysis GR designed the study, supervised data analysis, drafted and revised the manuscript.
